# Association of labor epidural analgesia use with exclusive breastfeeding up to six months: a online-based cross sectional survey in Jiaxing, China

**DOI:** 10.1186/s12884-022-05332-4

**Published:** 2022-12-28

**Authors:** Chun-Yan Fu, Li-Zhong Wang, Xue-Juan Tang, Feng Xia

**Affiliations:** 1 Department of Maternal Health, Jiaxing Maternity and Children Health Care Hospital, Affiliated Women and Children Hospital of Jiaxing University, Jiaxing, Zhejiang China; 2 Department of Anesthesiology, Jiaxing Maternity and Children Health Care Hospital, Affiliated Women and Children Hospital of Jiaxing University, Jiaxing, Zhejiang China

**Keywords:** Cross-sectional study, Epidural analgesia, Exclusive breastfeeding, Labor

## Abstract

**Background:**

The impact of labor epidural analgesia (LEA) on breastfeeding remains controversial. The aim of this study was to assess the relationship between LEA use and exclusive breastfeeding (EBF) up to 6 months.

**Methods:**

This was a cross-sectional survey on healthy mothers who had vaginal delivery with infants aged 7-12 months from seven maternal health WeChat groups in Jiaxing, China. Data including EBF status up to 6 months, maternal sociodemographic characteristics, LEA use in labor, breastfeeding supports during hospitalization and reasons for stopping EBF were collected using online self-administered questionnaires in October 2021. A multivariable logistic regression model was used to determine the potential association of LEA use with EBF up to 6 months by the adjusted odds ratio (AOR) and 95% confidence interval (CI).

**Results:**

Of a total of 537 surveyed mothers, 408 (76.0%) delivered with LEA and 398 (74.1%) exclusively breastfed their infants until 6 months. All mothers delivered in the hospitals with active breastfeeding policies. There was no statistical difference in the rate of EBF up to 6 months between mothers with and without LEA (73.8% versus 75.2%, *P* = 0.748). Multivariable logistic regression analysis indicated that only increased maternal age (AOR = 0.906, 95% CI 0.854-0.961, *P* = 0.001) and perceived insufficient breast milk (AOR = 0.129, 95% CI 0.082-0.204, *P* <  0.001) were associated with lower odds of EBF up to 6 months. The top three reasons for non-EBF were no or insufficient breast milk (41.7%), inability to breastfeed infants after return to work (27.3%), and maternal related factors (24.4%).

**Conclusions:**

LEA does not affect EBF up to 6 months. Other factors such as health education and breastfeeding-friendly hospital strategies may be much more important to breastfeeding outcomes compared to LEA use.

## Background

A wide range of benefits of breastfeeding for both infants and mothers in the short and long term are well-known. World Health Organization (WHO) recommends exclusively breastfeeding (EBF) of infants for the first 6 months after birth and sets as a target for 2025 to increase the EBF rate within 6 months up to at least 50% [1, 2]. However, the EBF rates remain suboptimal in many countries around the world, with the global EBF rate for infants 0-6 months old being about 37% [2]; in China, the EBF rate within 6 months was about 29.5% in 2018 [3].

Labor epidural analgesia (LEA) provides the most effective pain relief in labor as compared to other forms of analgesia, and has become one of the most common intrapartum interventions [4]. In China, LEA has been increasingly used over the past decade, with the rates of more than 50% in many obstetric hospitals [5]. With the increased use of LEA, its potential effects on breastfeeding have became one of the growing concerns for both health professionals and parturients. However, the impact of LEA on breastfeeding is controversial; some studies reported a lower incidence of breastfeeding postpartum in women who received LEA than those who did not, whereas others reported no relationship or even a positive association [6, 7]. Heterogeneity in study designs, breastfeeding indicators measured and the postpartum time-points of measurement may account for the discrepancies in these findings. For example, some studies measured EBF, while others measured mixed breastfeeding; the follow-up time-points also varied across studies, but most focused on short-term outcomes ranging from immediate after birth to 6-8 weeks postpartum [8]. In addition, other important factors which may impact on breastfeeding outcomes such as maternal educational level, work status, and breastfeeding supports available during hospitalization, have not been addressed in many studies regarding the impacts of LEA on breastfeeding [8]. Therefore, further efforts are warranted to analyze the relationship between LEA use and breastfeeding outcomes, in particular EBF up to 6 months. We therefore conducted this cross-sectional survey to evaluate the association between LEA use and the rate of EBF up to 6 months. Other relevant factors potentially affecting EBF up to 6 months were also assessed.

To improve maternal health care, we have established maternal health WeChat groups as part of traditional maternal health care since 2019 in Jiaxing, China. WeChat, the most widely used communication application in mainland China, supports one-on-one texting, group chats, multimedia sharing and more. Currently, a total of seven maternal health care WeChat groups, each having about 200-500 women, have been established in our area. In each WeChat group, a dedicated health worker is designated as the group manager who provides group education and individual counselling on maternal health including breastfeeding. The mode of WeChat groups also facilitates health workers to make the investigation of maternal health-related issues. This survey was therefore conducted based on the seven WeChat groups.

## Methods

This study was a part of a cross-sectional survey looking at the prevalence of EBF up to 6 months among the mothers from our seven maternal health WeChat groups in Jiaxing, China. This paper presented the results in relation to LEA use, EBF status up to 6 months and the associated factors. The survey was conducted in October 2021 using online self-administered questionnaires with the approval of the Research Ethics Committee of Jiaxing Maternity and Children Health Care Hospital (No. 2021-F-61). As it was an online survey, participants were fully informed in advance of the voluntary and anonymous nature of the survey, the requirement for written informed consent was waived by the Ethics Committee. The study is reported according to the Strengthening the Reporting of Observational Studies in Epidemiology (STROBE) guidelines [9].

The inclusion criteria were healthy mothers who had vaginal delivery with infants aged 7-12 months in our seven maternal health WeChat groups. The exclusion criteria were multiple gestations, premature delivery before 34 weeks, mastitis or prior breast surgery, major fetal congenital malformations, or any other problems that could affect breastfeeding practice.

The questionnaire was designed by the researchers and comprised dichotomous, multiple-choice or open-ended questions, including breastfeeding practice up to 6 months, maternal sociodemographic characteristics (maternal age, body mass index, ethnicity, educational level, marital status, and employment status), obstetric conditions (parity, gestational age, pregnancy complications, and birth weight), LEA use during labor, breastfeeding supports during hospitalization (skin-to-skin contact, rooming-in, and breastfeeding initiation), and perception of insufficient breast milk. There were three options for the breastfeeding practice up to 6 months (i.e. EBF, mixed feeding and no breastfeeding). EBF was defined as the infant only received breast milk without any additional food or drink, except for medicines, vitamins, and minerals, from birth to 6 months. Mothers who chose “mixed feeding” or “no breastfeeding” were grouped into non-EBF group and were asked to further report the reasons for non-EBF (open-ended and can be multiple).

The questionnaires were simultaneously released online by the WeChat group managers in the seven WeChat groups and all eligible mothers were invited to this survey. Each WeChat group manager who had been trained specifically for this survey was responsible for giving a full explanation to potential participants about the purpose of the survey, eligibility and exclusion criteria, and the voluntary and anonymity nature of the study including their right to withdraw from the study at any time, but once they submitted the questionnaire meant they agreed to participate in the study. If agreed, participates were asked to complete and submit the questionnaire online on their mobile phones within 2 weeks. During data collection, the group managers were also responsible for answering any questions raised by the participants regarding the questionnaire contents.

We did not specially investigate the mode and medications of LEA, because the surveyed mothers were unware of these details. However, under the guidance and supervision of a city-wide Labor Analgesia Quality Control Center, the LEA protocol used in the hospitals in Jiaxing is comparatively consistent, i.e. LEA is performed on the request of the woman during labor with bupivacaine (0.0625%) or ropivacaine (0.1%) combined with fentanyl (2 μg/mL) or sufentanil (0.5 μg/mL) administered by patient-controlled epidural analgesia (PCEA). Systemic opioids are not administered in labor. The analgesia protocols used were also confirmed by inquiring with the anesthesiologists of the hospitals where the surveyed mothers delivered after data collection.

### Statistical analysis

The study was powered to assess the primary outcome, i.e., the rate of EBF up to 6 months. A non-inferiority margin of 10% was used as we considered a decrease in EBF rate of no more than 10% in mothers delivering with LEA than those without was acceptable from a clinical perspective. Based on the results of a pre-survey, we hypothesized that the rate of EBF up to 6 months was 70% and LEA rate was 75% in mothers within our WeChat groups. Using PASS 15.0 software (NCSS, USA) and performing a one-sided *Z*-test (α = 0.025, β = 0.20), we calculated a required sample size of 321 for mothers with LEA and 107 for mothers without LEA.

Continuous variables were presented as median (range), categorical variables were presented as number and percentage. Normal distribution for continuous variables was checked using the Shapiro-Wilk test and visual plot inspection. As the continuous variables were not normally distributed, univariate analysis was performed using Mann-Whitney *U* test for continuous variables, and the *x*^2^ test or Fisher’s exact test for categorical variables to compare the differences between mothers with and without LEA, as well as between mothers with and without EBF. For the rate of EBF up to 6 months, we also used a non-inferiority margin of 10% difference with respect to the 95% CI. Additionally, a multivariable logistic regression model was used by the adjusted odds ratio (AOR) and 95% confidence interval (CI) to evaluate the association of LEA use and EBF up to 6 months, adjusting for potential confounders. Confounders were those variables that were significant in univariate analysis (*P* <  0.10). Before multivariable modeling, multicollinearity was assessed using the tolerance and variance inflation factor, with tolerance > 0.10 and variance inflation factor < 10.0 considered acceptable. Data were analyzed using SPSS version 19.0 for Windows (IBM Corp., Armonk, NY, USA). A *P* <  0.05 was considered as statistically significant.

## Results

**Fig. 1:**
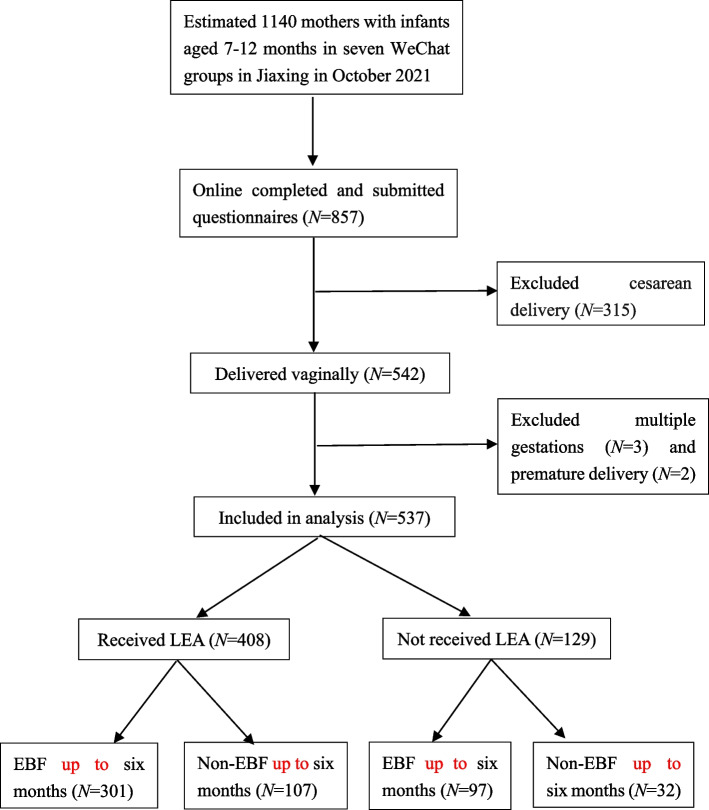
Flow diagram. *Abbreviation: EBF* exclusively breastfeeding, *LEA* labor epidural analgesia

It was estimated that there were about 1140 mothers with infants aged 7-12 months in seven WeChat groups at the survey time. A total of 857 mothers online completed and submitted the questionnaires, of them 542 (63.2%) delivered vaginally. Five mothers were excluded due to multiple gestations (*n* = 3) and premature delivery before 34 weeks (*n* = 2), leaving 537 mothers included in this study (Fig. 1). All mothers gave birth at seven public hospitals located in Jiaxing that provided LEA for 24 hours a day. Of the seven hospitals, five had Baby-Friendly Hospital Initiative (BFHI) certificates, but the other two also adopted breastfeeding policies similar to the BFHI hospitals. The majority of mothers had room-in (95.0%), skin-to-skin contact with their infants immediately after birth (88.3%), and breastfeeding initiation within the first hour of birth (71.5%).

Overall, 408 mothers (76.0%) delivered with LEA and 129 (24.0%) without LEA; 398 (74.1%) reported that they exclusively breastfed their infants until 6 months while 139 (25.9%) did not (Fig. 1). Data according to LEA use are presented in Table 1. There were no significant differences in variables between mothers with and without LEA, except for parity, i.e., primiparous mothers were more likely to choose LEA compared with multiparous mothers (*P* <  0.001). The rate of EBF up to 6 months was 73.8% (301/408) in mothers with LEA and 75.2% (97/129) in mothers without LEA with a difference of − 1.4% (95% CI − 7.6 to 9.4%), less than the10% margin of non-inferiority. Data according to EBF up to 6 months are presented in Table 2. Univariate analysis showed significant differences in maternal age, skin-to-skin contact after birth, breastfeeding initiation within the first hour, and perception of insufficient breast milk between mothers with and without EBF up to 6 months. No difference was found in LEA use between mothers with and without EBF up to 6 months (75.6% versus 77.0%, *P* = 0.748). Multivariable logistic regression analysis, using maternal age, skin-to-skin contact after birth, breastfeeding initiation within the first hour and perception of insufficient breast milk, together with LEA use as independent variables, indicated that only increased maternal age and perceived insufficient breast milk were significantly associated with lower odds of EBF up to 6 months (Table 3).

**Table 1:** Variables in mothers with and without LEA

Variables	LEA (***N*** = 408)	Non-LEA (***N*** = 129)	***P-***value
Maternal age (years), median (range)	29 (18-40)	29 (18-47)	0.104
BMI (kg·m^− 2^), median (range)	21.3 (15.4-38.5)	21.8 (16.2-38.9)	0.114
Educational level, n (%)			0.050
Junior high school or below	51 (12.5)	21 (16.3)	
High school	56 (13.7)	27 (20.9)	
College or above	301 (73.8)	81 (62.8)	
Marital status, n (%)			0.494
Married	400 (98.0)	125 (96.9)	
Unmarried	8 (2.0)	4 (3.1)	
Employment status, n (%)			0.397
Unemployed or part-time	160 (39.2)	56 (43.4)	
Full-time	248 (60.8)	73 (56.6)	
Parity, n (%)			< 0.001
Primiparas	301 (73.8)	65 (50.4)	
Multiparas	107 (26.2)	64 (49.6)	
Gestational age, n (%)			0.434
34-37 weeks	35 (8.6)	14 (10.9)	
> 37 weeks	373 (91.4)	115 (89.1)	
Pregnancy complications, n (%)			0.886
Yes	30 (7.4)	9 (7.0)	
No	378 (92.6)	120 (93.0)	
Postnatal complications, n (%)			0.730
Yes	8 (2.0)	3 (2.3)	
No	400 (98.0)	126 (97.7)	
Baby sex, n (%)			0.689
Male	210 (51.5)	69 (53.5)	
Female	198 (48.5)	60 (46.5)	
Birth weight, n (%)			0.419
≤ 2500 g	13 (3.2)	6 (4.7)	
> 2500 g	395 (96.8)	123 (95.3)	
Skin-to-skin contact, n (%)			0.225
Yes	364 (89.2)	110 (85.3)	
No	44 (10.8)	19 (14.7)	
Rooming-in, n (%)			0.666
Yes	385 (75.8)	123 (95.3)	
No	23 (5.6)	6 (4.7)	
Breastfeeding initiation after birth, n (%)			0.162
≤ 1 h	298 (73.0)	86 (66.7)	
> 1 h	110 (27.0)	43 (33.3)	
Perception of insufficient breast milk, n (%)			
Yes	159 (39.0)	51 (39.5)	0.909
No	249 (61.0)	78 (60.5)	
EBF up to 6 months, n (%)			0.748
Yes	301 (73.8)	97 (75.2)	
No	107 (26.2)	32 (24.8)	

Values are expressed as median (range) or numbers (percentages %)

*Abbreviation: BMI* body mass index, *EBF* exclusively breastfeeding, *LEA* labor epidural analgesia

**Table 2:** Variables in mothers with and without EBF up to 6 months

Variables	EBF (***N*** = 398)	Non-EBF (***N*** = 139)	***P-***value
Maternal age (years)	28 (18-39)	30 (23-47)	0.005
BMI (kg·m^−2^)	21.4 (16.0-38.5)	21.5 (15.4-38.9)	0.282
Educational level, n (%)			0.748
Junior high school or below	56 (14.1)	16 (11.5)	
High school	61(15.3)	22 (15.8)	
College or above	281(70.6)	101 (72.7)	
Marital status, n (%)			0.314
Married	387 (97.2)	138 (99.3)	
Unmarried	11 (2.8)	1 (0.7)	
Employment status, n (%)			0.235
Unemployed or part-time	166 (41.7)	50 (36.0)	
Full-time	232 (58.3)	89 (64.0)	
Parity, n (%)			0.713
Primiparas	273 (68.6)	93 (66.9)	
Multiparas	125 (31.4)	46 (33.1)	
Gestational age, n (%)			0.815
34-37 weeks	37 (9.3)	12 (8.6)	
> 37 weeks	361 (90.7)	127 (91.4)	
Pregnancy complications, n (%)			0.426
Yes	31 (7.8)	8 (5.8)	
No	367 (92.2)	131(94.2)	
Postnatal complications, n (%)			0.487
Yes	7 (1.8)	4 (2.9)	
No	391 (98.2)	135 (97.1)	
Baby sex, n (%)			0.877
Male	192 (48.2)	66 (47.5)	
Female	206 (51.8)	73(52.5)	
Birth weight, n (%)			0.112
≤ 2500 g	11 (2.8)	8 (5.8)	
> 2500 g	387 (97.2)	131 (94.2)	
LEA use, n (%)			0.748
Yes	301 (75.6)	107 (77.0)	
No	97 (24.4)	32 (23.0)	
Skin-to-skin contact, n (%)			0.019
Yes	359 (90.2)	115 (82.7)	
No	39 (9.8)	24 (17.3)	
Rooming-in, n (%)			0.277
Yes	379 (95.2)	129 (92.8)	
No	19 (4.8)	10 (7.2)	
Breastfeeding initiation after birth, n (%)			0.013
≤ 1 h	296 (74.4)	88 (63.3)	
> 1 h	102 (25.6)	51 (36.7)	
Perception of insufficient breast milk, n (%)			< 0.001
Yes	107 (26.9)	103 (74.1)	
No	291 (73.1)	36 (25.9)	

Values are expressed as median (range) or numbers (percentages %)

*Abbreviation: BMI* body mass index, *EBF* exclusively breastfeeding, *LEA* labor epidural analgesia

**Table 3:** Multivariable logistic regression of relevant variables with EBF up to 6 months

Variables	AOR	95% CI	***P-***value
Maternal age	0.906	0.854-0.961	0.001
Perceived insufficient breast milk	0.129	0.082-0.204	< 0.001
LEA use	0.845	0.500-1.421	0.520
Skin-to-skin contact	1.791	0.918-3.495	0.088
Breastfeeding initiation within the first hour	1.454	0.913-2.316	0.115

*Abbreviation: AOR* adjusted odds ratio, *CI* confidence interval, *EBF* exclusively breastfeeding, *LEA* labor epidural analgesia

In 139 mothers who failed to EBF up to 6 months, the top three reasons given for stopping EBF were no or insufficient breast milk (41.7%), inability to breastfeed their infants as needed after return to work (27.3%), and maternal related factors including the need for rest or being in worse mood (24.4%). Other reasons reported included no flexible nursing breaks at work (13.4%), the concern that breast milk alone was not sufficient for infant’s nutritional needs (10.1%), infant factors such as crying, illness and weight below the standard (9.4%), nipple and breast problems (6.5%), and no breastfeeding room or refrigerator for expressing or storing breast milk at the workplace (3.6%).

## Discussion

When assessing the impact of an intervention, it is preferable to perform a randomized controlled trial. Nevertheless, randomization between LEA and non LEA would be unethical, given the most effective labor analgesia provided by LEA [4]. Therefore, most of the studies examining the relationship between LEA and breastfeeding have been observational. In the current across-sectional survey of 537 mixed parity healthy mothers with vaginal delivery in our maternal health WeChat groups, we found that the rate of EBF up to 6 months was similar for mothers with and without LEA (73.8% versus 75.2%), suggesting that the use of LEA did not impact on EBF up to 6 months.

LEA is being increasingly used for pain relief in labor, but its effects on breastfeeding are still uncertain. A 2016 systematic review included 23 studies that investigated the association between LEA and breastfeeding showed conflicting results: no association in 10 studies, negative associations in 12 studies and positive association in one study [6]. Similarly, in a 2021 updated systematic review reporting on 15 studies, six studies reported no association, six studies reported negative association and three studies reported mixed results [7]. In only three prospective randomized studies to date that specially evaluate the effect of epidural fentanyl on breastfeeding, Beilin et al. [10] reported that mothers receiving a cumulative epidural fentanyl dose ≥150 μg were more likely to stop breastfeeding at 6 weeks postpartum compared with mothers receiving no fentanyl or a cumulative epidural fentanyl dose < 150 μg; in contrast, Wilson et al. [11] and Lee et al. [12] concluded that no association existed between epidural fentanyl dose and discontinuation of breastfeeding postpartum.

Different from most other studies [13–16], we used the rate of EBF up to 6 months as the primary outcome, as we considered it may be more clinically relevant and has been used as a main indicator of the success of breastfeeding in public health. We found no significant difference in EBF rate up to 6 months between mothers with and without LEA (73.8% versus 75.2%); in addition, multivariable logistic regression analysis also showed no association of LEA use with EBF up to 6 months after adjustment for other confounding variables. This result is consistent with some [13, 17] but in contrast to others [14, 15] of the recent observational studies. Additionally, in a current prospective observational study of a mixed-parity cohort, Orbach-Zinger et al. [16] reported that women with LEA was overall associated with reduced likelihood of breastfeeding at 6 weeks postpartum compared to those without, but they did not find this association in multiparous women with previous breastfeeding experience.

It is important, however, to note that the surveyed mothers in this study came from our maternal health WeChat groups, who may receive more breastfeeding education and would have a stronger intention to breastfeeding than those outside WeChat groups. Moreover, the surveyed mothers delivered at the hospitals which adopted the “Active Steps to Successful Breastfeeding”, including skin-to-skin contact, rooming-in with their infants, and breastfeeding initiation within the first hour after birth during hospitalization. As a result, it is not surprising that a significantly higher rate of EBF up to 6 months (74.1%) was found in this study compared to that reported in other study [3]. Our result therefore supports the view of other authors that, although the impact of LEA cannot be entirely ruled out, the environments strongly supportive of breastfeeding can offset the potential negative effects of LEA on breastfeeding [6, 16, 18]. In other words, breastfeeding education and hospitals policies of promoting breastfeeding are much more critical to breastfeeding outcomes than LEA use.

In our study setting, we found that only increased maternal age and perception of insufficient breast milk were negatively associated with EBF up to 6 months, especially that perceived insufficient breast milk significantly decreased the odds of EBF up to 6 months. We further investigated the reasons for why EBF was stopped during the first 6 months and, also, no or insufficient breast milk was the foremost one reported by the non-EBF mothers. This is consistent with previous studies [3, 19, 20]. However, in fact, only few mothers have physiological insufficient milk supply and most mothers can produce enough breastmilk to meet their infant’s demand [21]. As such, our result may imply the inadequate education and guidance provided by health workers on this issue. The next main reasons are inability to breastfeed their infants as needed after return to work and maternal related factors. Therefore, to promote EBF in China, the key is to improve breastfeeding education, especially related to insufficient breast milk, and provide a breastfeeding-friendly hospital and social environment.

This study has some limitations. First, our study only enrolled the mothers within maternal health WeChat groups, which could weaken the representativeness of the sample. Second, we did not investigate the exact type and amount of the medications used for LEA. However, as outlined in the methods, a comparatively consistent LEA protocol which represents currently the most commonly used labor analgesia was performed across the surveyed mothers. Third, the data on induction of labor, duration of labor, oxytocin augmentation and instrumental delivery, which have been reported to be associated with LEA [4] but can be potential confounders to the effects of LEA on breastfeeding outcomes [8], were unavailable due to the study design. Future studies in this area should consider these confounders. Fourth, because the mothers completed the questionnaire 6 months after delivery, the recall bias could not be avoided; in addition, the self-report nature of the study may have introduced a reporting bias. Finally, we did not monitor the trend of EBF rate over time. Although assessment of long-term breastfeeding outcomes is more clinically relevant, short-term outcomes should also be measured to explore the possible etiology of decreased breastfeeding rates.

## Conclusion

In summary, this survey shows that LEA has no impact on EBF up to 6 months in the mothers within our maternal health WeChat groups. Compared to LEA use, the health education and support from health workers along with breastfeeding-friendly hospital strategies may be much more important to breastfeeding outcomes.

## Data Availability

The datasets generated and/or analysed during the current study are not publicly available due to privacy and ownership but are available from the corresponding author on reasonable request.
